# Association of albumin trajectories and cumulative exposure with in-hospital mortality in acute pancreatitis: a retrospective cohort study

**DOI:** 10.1097/JS9.0000000000003354

**Published:** 2025-09-19

**Authors:** Yaoyu Zou, Maobin Kuang, Shixuan Xiong, Xin Xu, Xueyang Li, Ling Ding, Cong He, Nianshuang Li, Huajing Ke, Xin Huang, Yupeng Lei, Huifang Xiong, Wenhua He, Lingyu Luo, Liang Xia, Nonghua Lu, Jianhua Wan, Yin Zhu

**Affiliations:** Department of Gastroenterology, Jiangxi Provincial Key Laboratory of Digestive Diseases, Jiangxi Clinical Research Center for Gastroenterology, Digestive Disease Hospital, The First Affiliated Hospital, Jiangxi Medical College, Nanchang University, Nanchang, Jiangxi, China

**Keywords:** acute pancreatitis, albumin trajectory, cumulative albumin exposure, in-hospital mortality

## Abstract

**Background:**

Hypoalbuminemia is common in acute pancreatitis (AP) and associated with poor outcomes, but its cumulative and dynamic impacts are not well characterized.

**Methods:**

This study included 3,214 AP patients (2005–2023). Cumulative albumin exposure (CumALB) was calculated over the first 7 days using the trapezoidal method. Latent class mixed modeling (LCMM) identified distinct albumin trajectories. Multivariable logistic regression and restricted cubic splines (RCS) assessed associations between CumALB and in-hospital mortality. Kaplan–Meier curves compared survival across trajectories. A simplified nomogram was developed using predictors selected via Boruta and LASSO algorithms, with performance assessed by AUC, calibration, and decision curves. Validation was conducted in three cohorts: the MIMIC-IV database (*n* = 514), the eICU-CRD database (*n* = 211), and the local cohort from 2024 (*n* = 880).

**Results:**

Patients in the lowest CumALB tertile had significantly higher mortality (11.0%) than those in the highest tertile (1.3%, *P* < 0.001). Each 1-SD increase in CumALB reduced mortality risk by 42% (adjusted OR = 0.58, 95% CI: 0.44–0.76, *P* < 0.001), with RCS confirming a linear inverse relationship. CumALB outperformed single-day albumin in mortality prediction. LCMM identified four trajectories; low-stable (LS-T1) had highest mortality (17.7%), whereas low-increasing (LI-T4) had lower mortality (8.6%). Compared with LS-T1, high-stable (HS-T2) and LI-T4 groups had reduced risks (adjusted OR = 0.16 and 0.49, respectively; *P* < 0.05). The CumALB-based nomogram achieved an AUC of 0.836 in the training set (70%) and 0.864 in the internal test set (30%), outperforming APACHE II (AUC = 0.76), SIRS (AUC = 0.72), Ranson (AUC = 0.72), and BISAP (AUC = 0.80). Validation was conducted using three independent cohorts: the MIMIC-IV cohort (AUC = 0.631), the eICU-CRD cohort (AUC = 0.681), and the local cohort from 2024 (AUC = 0.844). Sensitivity analyses further confirmed the robustness of these results.

**Conclusion:**

CumALB may provide a useful measure of the cumulative burden of hypoalbuminemia in patients with acute pancreatitis, and albumin trajectories could help capture its dynamic changes. The CumALB-based predictive model demonstrated good performance in predicting mortality risk, potentially assisting clinicians in early risk stratification and clinical decision-making for high-risk patients within heterogeneous populations. However, prospective validation is warranted before clinical implementation.

**HIGHLIGHTS**

- We introduced cumulative albumin exposure (CumALB) and dynamic trajectory modeling to comprehensively quantify the prognostic value of hypoalbuminemia in acute pancreatitis (AP).
- Leveraging a large patient cohort, CumALB demonstrated a robust linear inverse association with in-hospital mortality, significantly outperforming single-day albumin measurements and established prognostic scoring systems (APACHE II, SIRS, Ranson, and BISAP).
- Four distinct albumin trajectories were identified, quantitatively linking dynamic albumin fluctuations to mortality risk: patients with a low-stable trajectory (LS-T1) exhibited the highest mortality rate (17.7%), whereas those with a low-increasing trajectory (LI-T4) had a notably lower mortality rate (8.6%).
- A simplified CumALB-based nomogram, incorporating only four routine clinical variables (CumALB, pulse, WBC, and creatinine), demonstrated excellent predictive performance (AUC = 0.836), offering clinicians a practical tool for the rapid identification of AP patients at mid-to-late in-hospital mortality risk. External validation using three independent cohorts—the MIMIC-IV cohort (AUC = 0.631), the eICU-CRD cohort (AUC = 0.681), and a prospectively maintained local cohort from 2024 (AUC = 0.844)—confirmed the stability and clinical utility of the model.

## Introduction

Acute pancreatitis (AP) is a common and increasingly prevalent gastrointestinal disorder worldwide, with approximately 20%–30% of patients progressing to severe acute pancreatitis (SAP)^[1]^. SAP is characterized by persistent organ failure (POF) lasting more than 48 hours, with a mortality rate as high as 30%^[2,3]^. Previous studies have shown that early fluid resuscitation, rational antibiotic use, nutritional support, and etiological treatment are critical interventions that determine clinical outcomes in SAP^[4,5]^. Therefore, timely and accurate identification of high-risk patients, along with dynamic assessment of disease severity, is essential to optimize treatment strategies and improve prognosis.

However, widely used clinical scoring systems—such as the Acute Physiology and Chronic Health Evaluation II (APACHE II), Bedside Index of Severity in Acute Pancreatitis (BISAP), and Sequential Organ Failure Assessment (SOFA)—are primarily based on static indicators at admission (e.g., vital signs and laboratory results). These models often fail to capture evolving pathophysiological changes throughout disease progression and lack quantitative evaluation of dynamic trends in key biomarkers, which limits their early warning capability^[6]^.

Serum albumin (ALB), a negative acute-phase protein synthesized by the liver, synthesized by the liver, plays essential roles in maintaining colloid osmotic pressure, exerting antiinflammatory and antioxidant effects, and serving as a key indicator of nutritional status.^[7–9]^. Hypoalbuminemia is common in AP patients and is strongly associated with POF, severe disease progression, and increased mortality^[10–12]^. However, most existing evidence relies on single-time-point measurements, with limited data on the longitudinal trajectory of ALB levels throughout disease progression. The Hungarian Pancreatic Study Group demonstrated that, among AP patients, the lowest in-hospital serum ALB level outperformed admission values in predicting in-hospital mortality (AUC = 0.747 vs. 0.660)^[12]^. In sepsis, persistently low ALB levels over time were independently linked to worse outcomes^[13]^. These findings highlight the prognostic value of dynamic ALB monitoring, suggesting that longitudinal trajectories could enable more accurate risk stratification than static single-point measurements.

In summary, despite the increasing recognition of dynamic ALB monitoring as clinically valuable in various critical illnesses, comprehensive research specifically focusing on AP remains limited. Therefore, based on a large longitudinal cohort, this study investigated cumulative albumin exposure (CumALB) and dynamic ALB trajectory phenotypes in patients with AP. Our objectives were to quantitatively characterize the dose–response relationship between time-weighted CumALB burden and in-hospital mortality, identify distinct dynamic ALB trajectory subtypes with significant prognostic implications, and thus provide evidence supporting the transformation of AP management from empirical treatment toward a precision medicine approach informed by real-time dynamic clinical data, ultimately improving patient outcomes and reducing mortality. This cohort study has been reported in line with the strengthening the reporting of cohort, cross-sectional and case-control studies in surgery (STROCSS) guidelines^[14]^.

## Methods

### Study design and population

**Figure 1.**
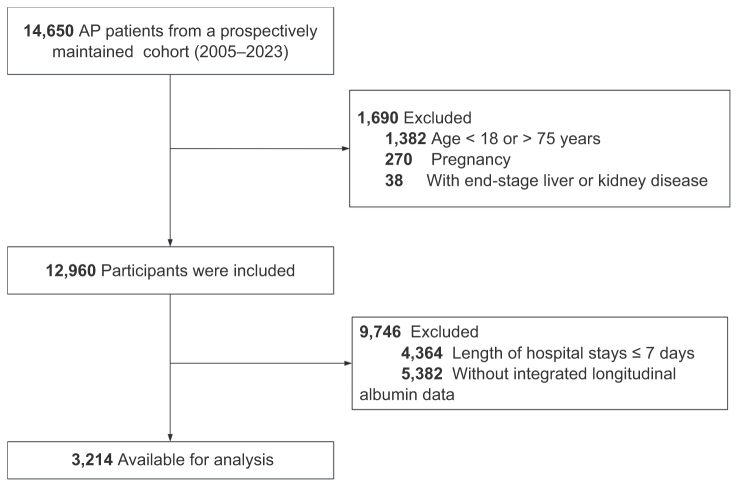
Flowchart of the screening and enrollment of study participants. AP, Acute pancreatitis.

This study comprised a retrospective cohort (Fig. 1) and three validation cohorts (Supplemental Digital Content Figs S1–S3, available at, http://links.lww.com/JS9/F9). The retrospective cohort was derived from a continuously updated inpatient database maintained by the Department of Gastroenterology at the First Affiliated Hospital of Nanchang University since 2005. From January 2005 to 14 June 2023, 650 hospitalized patients diagnosed with AP were identified. Based on the study design, patients were excluded if they met any of the following criteria: age <18 or >75 years (*n* = 1,382), pregnancy (*n* = 270), end-stage liver or renal failure (*n* = 38), hospital stay ≤7 days (*n* = 4364), or patients lacking albumin measurements at two or more of the five required time points(*n* = 5382). Ultimately, 3214 patients were included in the primary analysis. All included patients met the 2012 revised Atlanta Classification criteria for AP diagnosis^[2]^, which requires at least two of the following: (1) persistent upper abdominal pain, (2) serum amylase or lipase levels ≥3× the upper limit of normal, or (3) radiographic evidence of pancreatitis. The primary outcome measure of the current study was in-hospital mortality, defined as death occurring from any cause during hospitalization.

The validation cohorts were derived from three distinct sources: the MIMIC-IV database (Version 3.0), the eICU-CRD database, and the local cohort (2024). Employing inclusion and exclusion criteria consistent with those applied to the retrospective local cohort, we identified 514 ICU-admitted AP patients from MIMIC-IV, 211 patients from eICU-CRD, and 880 patients from the local cohort (2024), forming the internal and external validation sets. In the MIMIC-IV and eICU-CRD databases, subjects were identified using discharge diagnosis codes ICD-9 (577.0) and ICD-10 (K85.92), in line with methods established in prior research^[15,16]^.

This study was approved by the Ethics Committee of the First Affiliated Hospital of Nanchang University (Approval No. 2011001). All data were anonymized to protect patient privacy. The principal investigator, Dr. Shixuan Xiong, completed the Collaborative Institutional Training Initiative (CITI) course and holds certification (Certificate No. 58279303) for authorized access to the MIMIC-IV database. The study adhered to the ethical principles of the Declaration of Helsinki (2013 revision). As a retrospective analysis, informed consent was waived by the Ethics Committee.

### Data collection and variables

Demographic, clinical, laboratory, and outcome data were systematically retrieved from our institution’s electronic medical records and the publicly available MIMIC-IV and eICU-CRD databases. Two independent investigators extracted data using standardized data collection forms, with discrepancies resolved by consensus from a senior investigator.

Demographic variables included age, sex, weight, and body mass index (BMI). Comorbidities encompassed hypertension, hyperlipidemia, diabetes, coronary artery disease, and chronic kidney disease. Lifestyle factors included smoking status and alcohol use. Clinical characteristics recorded were AP etiology (biliary, hypertriglyceridemia, alcohol-related, or other), symptom onset to admission interval, hospital length of stay, intensive care unit (ICU) admission status and duration, and in-hospital death. Vital signs documented included daily temperature, heart rate, respiratory rate, systolic, and diastolic blood pressures. Laboratory parameters included white blood cell count (WBC), neutrophil count (NEU), glucose (GLU), calcium (Ca), triglycerides (TG), creatinine (Cr), hemoglobin (Hb), total bilirubin (TBIL), C-reactive protein (CRP), and serum albumin (ALB) values recorded on Days 1, 2, 3, 5, and 7. Severity scores comprised APACHE II, SIRS, Ranson, and BISAP. Detailed therapeutic interventions included early albumin infusion within 72 hours, mechanical ventilation, continuous renal replacement therapy (CRRT), and percutaneous catheter drainage (PCD).

The primary exposure variable was the CumALB during the first 7 days of hospitalization, calculated using the trapezoidal method to reflect the integrated temporal trend of ALB changes. The formula is as follows: $CumALB=\sum_{i=1}^{n-1}\left(\frac{ALB_{i}+ALB_{i+1}}{2}\times \Delta t_{i}\right)$. where ALBiand $ALB_{i+1}$ are the ALB levels on day i and day i+1, respectively, and $\Delta t_{i}$ is the time interval between measurements (in days)^[17,18]^. For patients who did not return for day_2_, *CumALB* was calculated as (*ALB*_1_ + *ALB*_3_)/2 × time_3-1_, as previously described^[19]^.

### Serum albumin measurement

For all study participants, peripheral venous blood samples were collected as required following admission, specifically for the measurement of albumin and other biochemical parameters. Blood samples were drawn into standard vacuum collection tubes for biochemical assays, centrifuged, and sent immediately for analysis. Serum ALB levels were routinely measured by the clinical laboratory using the bromocresol green (BCG) dye-binding method (Autobio, China) according to the manufacturer’s instructions. Analyses were performed using the Roche Cobas 8000 series automatic biochemical analyzer at a wavelength of 600–630 nm, with a reference range of 35–55 g/L. The assay demonstrated excellent reproducibility and accuracy, with good linearity across an albumin concentration range of 0–70 g/L (correlation coefficient *R*^2^ > 0.99), an interassay coefficient of variation (CV) ≤ 2%, and a limit of detection (LoD) of 3.5 g/L. The presence of common interfering substances did not significantly affect assay results if their concentrations remained within the following limits: bilirubin ≤400 µmol/L, hemoglobin ≤5 g/L, lipemia <1.60%, and ascorbic acid ≤0.5 g/L. All analyses were conducted using the same laboratory platform to ensure data consistency.

### Handling of missing data

For variables with missing data, tailored imputation strategies were applied based on the proportion of missing values. Variables with >5% missing data (e.g., BMI [17.5%], CK [11.6%], GLU [8.8%]) were addressed via multiple imputation to minimize bias and enhance statistical validity^[20,21]^. Variables with <0.2% missingness (e.g., pulse, respiratory rate, temperature) were imputed using single imputation (e.g., median or mode). A summary of missing data is provided in Supplemental Digital Content Table S1, available at http://links.lww.com/JS9/F9. The primary analysis was conducted on the original dataset, while sensitivity analyses utilized the imputed dataset to evaluate the robustness of the association between CumALB and in-hospital mortality.

### Latent class mixed modeling (LCMM) analysis

Latent class mixed modeling (LCMM) is a robust statistical technique that identifies patient subgroups with heterogeneous longitudinal outcome trajectories^[22]^. It integrates mixed-effects modeling and latent class analysis, capturing individual-level temporal changes through random effects and classifying patients into latent classes with similar longitudinal patterns, thus revealing population heterogeneity^[23]^. Each latent class follows a distinct polynomial trajectory, and individuals retain class-specific random effects, which improves model fit compared with traditional growth models^[24]^. Model performance was evaluated using convergence status, Bayesian Information Criterion (BIC), and a minimum class size threshold (≥ 5%)^[25]^. Recent studies have applied LCMM to identify novel sepsis phenotypes^[26]^. In this study, ALB trajectories were modeled using albumin measurements from at least three of the five designated time points (Days 1, 2, 3, 5, and 7)^[27,28]^.

### Model development and validation

To predict the risk of in-hospital mortality among patients with AP, this study developed and validated a logistic regression nomogram using a combined Boruta-LASSO feature-selection approach. Initially, candidate clinical variables were screened in the training set (comprising 70% of the total data) using the Boruta algorithm—a random forest-based wrapper method. Boruta robustly identified truly outcome-related variables by iteratively comparing *Z*-scores of real variables with those of randomly generated “shadow features,” retaining only variables whose importance consistently exceeded random levels^[29]^. Subsequently, variables selected by Boruta underwent further refinement using Least Absolute Shrinkage and Selection Operator (LASSO) regression with tenfold cross-validation to determine the optimal regularization parameter (*λ*). Only variables with nonzero coefficients were retained, thereby effectively controlling model complexity and reducing the risk of overfitting^[30]^.

Integrating Boruta and LASSO algorithms provided a complementary approach to selecting biomarkers predictive of AP in-hospital mortality. Boruta’s iterative elimination of noninformative features enhanced the robustness of feature selection, whereas LASSO’s shrinkage properties effectively regulated model complexity. This combined method alleviated overfitting, a common challenge in high-dimensional data analyses. Moreover, previous research indicates that integrated feature-selection methods typically achieve superior classification accuracy compared to single-method approaches^[31]^. By integrating these techniques, the strategy facilitated a more comprehensive and reliable identification of biomarkers related to in-hospital mortality among AP patients.

Given the study’s aim of providing clinicians with a concise, reliable, and interpretable predictive tool for mortality risk, logistic regression was employed to create the AP mortality prediction model, visualizing each risk factor’s contribution through a nomogram. Model performance was assessed using the area under the receiver operating characteristic (ROC) curve (AUC) and Hosmer–Lemeshow calibration curves, evaluating discrimination and predictive consistency, respectively. Clinical utility was further appraised using decision curve analysis (DCA), assessing the practical benefit of the model in clinical decision-making. Additionally, to enhance interpretability, SHapley Additive exPlanations (SHAP) were applied^[32]^. SHAP values were computed for each feature across all patients, quantifying their importance in prediction outcomes, with higher absolute SHAP values indicating greater influence on model predictions.

### Statistical analysis

All baseline characteristics were summarized as means (± standard deviation), medians (interquartile range), or counts (percentages) based on data distribution. Between-group comparisons were performed with Student’s *t*-test or Mann–Whitney *U* test for continuous variables and *χ*^2^ test for categorical variables. Multicollinearity among covariates was evaluated using linear regression models, with a variance inflation factor (VIF) > 5 indicating collinearity; collinear variables were excluded from final models^[33]^ (Supplemental Digital Content Table S2, available at, http://links.lww.com/JS9/F9).

The associations of CumALB exposure and ALB trajectory classes with in-hospital mortality were analyzed using multivariable logistic regression. Three progressively adjusted models were constructed: Crude model: unadjusted; Model 1: adjusted for demographic factors (age, sex, BMI); Model 2: additionally adjusted for lifestyle factors (smoking, alcohol), comorbidities (hypertension, hyperlipidemia, and diabetes), and basic vital signs (temperature, heart rate, respiratory rate, and systolic/diastolic blood pressure); Model 3: further adjusted for key laboratory markers (NEU, platelets, CK, TBIL, GLU, and TG). Nonlinear relationships between CumALB and mortality were explored using restricted cubic splines (RCS) with three knots, and likelihood ratio tests were conducted to assess deviations from linearity. RCS is a flexible statistical method widely employed in regression modeling to fit nonlinear relationships by connecting cubic polynomial segments at predetermined knots, while enforcing linear constraints at the boundaries. This approach achieves flexibility in capturing complex relationships and simultaneously reduces the risk of unstable extrapolation caused by overfitting^[34,35]^. Kaplan–Meier survival analysis was conducted to examine the association between early ALB trajectory classes and mortality, with log-rank tests for between-group comparisons. ROC curves, area under the curve (AUC), sensitivity, specificity, and optimal thresholds were derived to compare model performance.

Several sensitivity analyses were conducted to confirm robustness: (1) Replaced baseline temperature and respiratory rate with their 7-day cumulative values; (2) Repeated regression analyses in a multiply imputed dataset for variables with minor missingness; (3) Propensity score matching (PSM; 1:1 ratio) with the LS-T1 trajectory class as the exposure group to balance baseline characteristics; (4) Evaluated associations based on 72-hour CumALB and admission-day albumin; (5) Adjusted the primary model for therapeutic interventions (albumin infusion within 72 h, CRRT, PCD, mechanical ventilation); (6) Conducted temporal sensitivity analysis excluding early-phase patients (2005–2014); (7) Performed analysis excluding patients with immunosuppressive diseases or active malignancies; (8) Conducted subgroup analyses stratified by age, sex, BMI, etiology, comorbidities, disease severity, APACHE II, and SIRS scores to test for interaction effects.

All analyses were performed in R software (v4.2.2). Statistical significance was defined as a two-sided *P* < 0.05.

## Results

### Baseline characteristics and outcomes by cumulative albumin exposure

**Figure 2.**
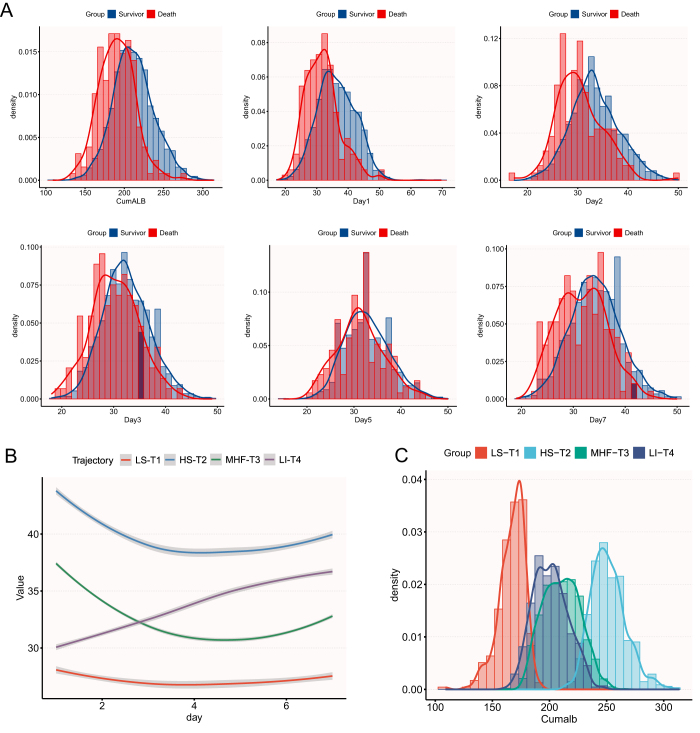
Cumulative albumin exposure and dynamic albumin trajectories in patients with acute pancreatitis. **A**. The distribution of CumALB and ALB at different time points (Day 1, Day 3, Day 5, Day 7) in mortality of AP. Red curves and bars indicate nonsurvivors; blue curves and bars indicate survivors. **B.** ALB trajectories during the first 7 days after admission, identified using LCMM. **C.** Density distributions of CumALB across the four ALB trajectory groups. ALB, albumin; CumALB, cumulative albumin; LCMM, latent class trajectory modeling; LS-T1, low–stable trajectory; HS-T2, high–stable trajectory; MHF-T3, mid–high–fluctuating trajectory; LI-T4, low-increasing trajectory.

A total of 3214 AP patients were included to evaluate associations between early CumALB and clinical outcomes. Given the large number of excluded patients due to insufficient hospitalization duration or missing longitudinal albumin data, we compared baseline characteristics between the excluded group (*n* = 9746) and the included group (*n* = 3214) to assess potential selection bias (Supplemental Digital Content Table S3, available at, http://links.lww.com/JS9/F9). Results indicated minimal differences between the two groups across demographic and most clinical variables, with standardized mean differences (SMD) consistently less than 0.2, suggesting no substantial imbalance overall. Baseline characteristics stratified by CumALB tertiles are presented in Supplemental Digital Content Table S4, available at http://links.lww.com/JS9/F9. Significant differences were observed across tertiles in demographics (lowest tertile: older age, higher female proportion), etiology (higher biliary AP prevalence), comorbidities (e.g., hypertension, cardiovascular diseases), and laboratory parameters. Inflammatory markers (WBC, NEU) and severity scores (SIRS, APACHE II) were most elevated in the lowest CumALB tertile (all *P* < 0.001). Probability density curves (Fig. 2A) further highlighted CumALB’s discriminatory power compared to single-time-point ALB measurements.

In the lowest CumALB tertile, organ failure incidence was 66.9%, persistent multiple organ failure (PMOF) 19.4%, and in-hospital mortality 11.0%—all significantly higher than the highest tertile (34.5%, 5.0%, and 1.3%, respectively; *P* < 0.001; Supplemental Digital Content Table S5, available at, http://links.lww.com/JS9/F9). Complications (e.g., WON, IPN), severe AP (SAP), and prolonged hospitalization also increased with declining CumALB levels (all *P* < 0.001). Supplemental Digital Content Figs S4–S5 (available at, http://links.lww.com/JS9/F9) demonstrate consistent CumALB associations across outcomes and etiologies.

### Associations of cumulative albumin exposure with mortality risk and predictive performance

**Figure 3.**
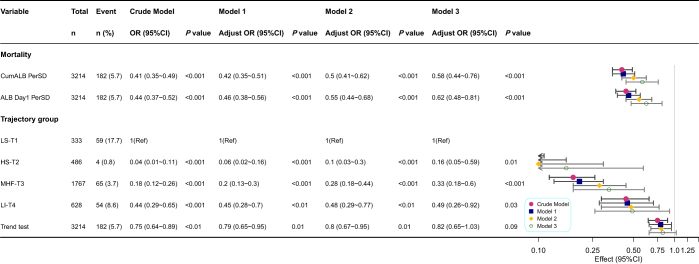
Association of cumulative albumin exposure and albumin trajectories with in-hospital mortality in acute pancreatitis. ALB, albumin; CumALB, cumulative albumin; OR, odds ratio; CI, confidence interval; SD, standard deviation; BMI, body mass index; SBP, systolic blood pressure; DBP, diastolic blood pressure; NEU, neutrophil count; PLT, platelet count; CK, creatine kinase; TBIL, total bilirubin; GLU, glucose; TG, triglyceride; Ref, reference group. Crude model was unadjusted. Model 1 was adjusted for sex, age, and BMI. Model 2 was further adjusted for smoking history, alcohol consumption, history of hypertension, hyperlipidemia, diabetes, temperature, pulse, respirations, SBP, and DBP. Model 3 was adjusted for the same variables as Model 2, plus NEU, PLT, CK, TBIL, GLU, and TG. The LS-T1 group served as the reference. The trend test was performed by modeling the trajectory group as an ordinal variable.

**Figure 4.**
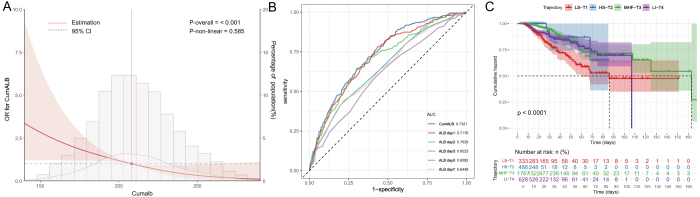
**A.** Predictive and prognostic value of cumulative albumin and albumin trajectories in acute pancreatitis. Relationship between CumALB and in-hospital mortality estimated using a RCS model with 3 knots. **B.** ROC curves comparing the predictive performance of CumALB and ALB levels on Days 1, 2, 3, 5, and 7 for in-hospital mortality. AUC values are shown in the legend. **C.** Kaplan–Meier survival curves stratified by ALB trajectory groups identified by LCMM. Log-rank test was used to compare survival distributions. CumALB, cumulative albumin; ALB, albumin; OR, odds ratio; CI, confidence interval; RCS, restricted cubic spline; ROC, receiver operating characteristic; AUC, area under the curve; LCMM, latent class trajectory modeling; LS-T1, low–stable trajectory; HS-T2, high–stable trajectory; MHF-T3, mid–high-fluctuating trajectory; LI-T4, low–increasing trajectory.

Multivariable logistic regression demonstrated a dose–response inverse association between CumALB and in-hospital mortality (Fig. 3). In the unadjusted model, each 1-SD increase in CumALB was linked to a 59% reduction in mortality risk (OR = 0.41, 95% CI: 0.35–0.49; *P* < 0.001). This relationship persisted after full adjustment (Model 3: OR = 0.58, 95% CI: 0.44–0.76; *P* < 0.001). RCS analysis confirmed a linear inverse association between CumALB and mortality (Fig. 4A). CumALB showed superior discrimination for mortality in AP patients compared to single-day measurements, achieving the highest AUC of 0.732 (95% CI: 0.697–0.767). Day 1 ALB ranked second (AUC = 0.718, 95% CI: 0.681–0.755) (Fig. 4B, Supplemental Digital Content Table S6, available at, http://links.lww.com/JS9/F9).

### Association of early albumin infusion with CumALB

To examine the association between early albumin infusion and CumALB, we performed a propensity score–matched comparison among patients exhibiting low albumin trajectories (combined LS-T1 and LI-T4). After matching for demographics, comorbidities, vital signs, and laboratory parameters, survivors who received early albumin infusion within the first 3 days demonstrated significantly higher early CumALB levels (median: 198 vs. 186; *P* < 0.01), whereas no significant difference was observed among nonsurvivors (183.9 vs. 176.7; *P* = 0.43) (Supplemental Digital Content Fig. S6, available at, http://links.lww.com/JS9/F9). Furthermore, patients in the overall cohort who received early albumin infusion exhibited higher early CumALB compared to those without infusion. As anticipated, higher early CumALB was significantly associated with the administration of albumin (adjusted OR = 1.43, 95% CI: 1.17–1.76; Supplemental Digital Content Table S7, available at, http://links.lww.com/JS9/F9).

### Albumin trajectories and their characteristics

To complement CumALB and identify distinct ALB trajectory patterns related to mortality, the four-class LCMM was selected based on superior BIC/SABIC and convergence compared with 2–5 class alternatives, offering the best balance of model fit and clinical interpretability (Fig. 2B; Supplemental Digital Content Table S8, available at, http://links.lww.com/JS9/F9). Class sizes were ~ 15.1%, 10.4%, 55.0%, and 19.5%.

***T1 (Low-stable, LS-T1)***—Patients in this trajectory demonstrated persistently low serum albumin levels from admission through the entire observation period without notable improvement. This pattern suggests a state of sustained systemic inflammation, severe malnutrition, and a high risk of potential organ dysfunction, typically indicating a poorer clinical prognosis.

***T2 (High-stable, HS-T2)***—Patients exhibited serum albumin levels consistently above the normal threshold upon admission, with only minor fluctuations observed around days 3–4. This stable, higher-level trajectory indicates well-maintained hepatic synthesis and nutritional status, generally implying low complication risk. Hence, it serves as a baseline reference for a low-risk subgroup.

***T3 (Moderate-high fluctuating, MHF-T3)***—Patients started with moderate-to-high serum albumin levels upon admission, experienced a marked acute decline reaching a nadir around day 4, followed by a gradual recovery to near-baseline levels, forming a characteristic “U-shaped” trajectory. This pattern suggests preserved hepatic synthetic reserve and robust immunological and inflammatory regulation, allowing effective adaptation to acute physiological stress, with relatively favorable outcomes for most patients.

***T4 (Low-increasing, LI-T4)***—Patients in this class initially presented with low albumin levels upon admission but showed a steady, progressive increase from day 2 onward, approaching normal levels by the end of the observation period. This trajectory indicates a favorable response to clinical intervention and gradual recovery of hepatic synthetic function. However, due to the initial hypoalbuminemic state, patients remain at some clinical risk and require continued monitoring to prevent potential recurrence of complications.

CumALB distribution varied significantly across trajectories (*P* < 0.001), with the highest levels in HS-T2 and the lowest in LS-T1 (Fig. 2C). Baseline characteristics differed markedly: LS-T1 patients were older, had higher biliary etiology prevalence, and more severe inflammation (e.g., elevated WBC, CRP), while HS-T2 patients exhibited stable profiles (Table 1).Trajectory classes also strongly differentiated outcomes (Table 2). The LS-T1 group exhibited the worst outcomes, with an organ failure rate of 68.8% and an in-hospital mortality of 17.7%. Although the LI-T4 group started with low ALB levels, their upward trend over time was associated with improved outcomes compared to LS-T1 (mortality 8.6%, *P* < 0.001), though still significantly worse than the HS-T2 group (mortality 0.8%, *P* < 0.001). Compared with LS-T1, the HS-T2 group had the lowest adjusted mortality risk (OR = 0.16, 95% CI: 0.05–0.59), and the LI-T4 group also had significantly reduced risk (OR = 0.49, 95% CI: 0.26–0.92) (Fig. 3). Kaplan–Meier survival analysis further demonstrated substantial survival differences across trajectory classes, with the LS-T1 group showing significantly lower survival probabilities compared to the other three groups (Fig. 4C, log-rank test *P* < 0.001).

**Table 1:** Comparisons of baseline characteristics of the AP patients with different ALB trajectory

Variables	Total (*n* = 3214)	LS-T1 (*n* = 333)	HS-T2 (*n* = 486)	MHF-T3 (*n* = 1767)	LI-T4 (*n* = 628)	*P*
General characteristics
Male, *n* (%)	2018 (62.8)	191 (57.4)	329 (67.7)	1136 (64.3)	362 (57.6)	<0.001
Age (years)	49.2 ± 13.9	52.3 ± 13.4	43.9 ± 12.9	49.6 ± 14.0	50.5 ± 13.9	<0.001
BMI	24.5 ± 4.6	23.7 ± 5.5	25.1 ± 4.8	24.5 ± 4.4	24.3 ± 4.2	0.001
Etiology, *n* (%)
Biliary	1500 (46.7)	180 (54.1)	189 (38.9)	858 (48.6)	273 (43.5)	
HTG	1079 (33.6)	85 (25.5)	198 (40.7)	592 (33.5)	204 (32.5)	
Alcoholism	168 (5.2)	24 (7.2)	25 (5.1)	81 (4.6)	38 (6.1)	
Others	467 (14.5)	44 (13.2)	74 (15.2)	236 (13.4)	113 (18)	
Smoking, *n* (%)	960 (29.9)	97 (29.1)	153 (31.5)	527 (29.8)	183 (29.1)	0.837
Drinking, *n* (%)	1037 (32.3)	99 (29.7)	161 (33.1)	569 (32.2)	208 (33.1)	0.715
Cardiovascular disease, *n* (%)	647 (20.3)	74 (22.4)	77 (16)	361 (20.6)	135 (21.6)	0.066
Hypertensive disease, *n* (%)	650 (20.2)	76 (22.8)	81 (16.7)	360 (20.4)	133 (21.2)	0.134
History of HTG	349 (10.9)	30 (9)	72 (14.8)	180 (10.2)	67 (10.7)	0.02
Diabetes, *n* (%)	475 (14.8)	46 (13.8)	68 (14)	277 (15.7)	84 (13.4)	0.458
Vital signs
Temperature, °C	37.1 ± 0.8	37.4 ± 0.9	36.8 ± 0.6	37.0 ± 0.7	37.3 ± 0.8	<0.001
Pulse, times/min	98.0 ± 20.2	105.4 ± 20.6	91.6 ± 18.6	97.3 ± 20.1	101.1 ± 20.0	<0.001
Respirations, times/min	22.4 ± 5.5	23.6 ± 6.2	21.0 ± 4.3	22.0 ± 5.1	23.8 ± 6.7	<0.001
SBP, mmHg	128.4 ± 19.9	126.8 ± 21.0	129.0 ± 18.4	129.6 ± 19.7	125.5 ± 20.5	<0.001
DBP, mmHg	81.2 ± 14.6	78.3 ± 14.7	83.5 ± 14.9	82.2 ± 14.2	78.1 ± 14.5	<0.001
Laboratory parameters
WBC, 10^9^/L	12.8 ± 5.9	13.5 ± 6.9	10.8 ± 4.8	13.1 ± 5.8	13.1 ± 6.1	<0.001
NEU, 10^9^/L	10.8 ± 5.8	11.6 ± 6.6	8.7 ± 4.7	11.1 ± 5.7	11.1 ± 5.9	<0.001
Hb, g/L	133.1 ± 28.8	114.7 ± 27.3	143.1 ± 22.7	138.2 ± 27.5	120.8 ± 29.2	<0.001
HbA1c, %	6.8 ± 2.0	6.4 ± 1.8	7.1 ± 2.2	6.9 ± 2.1	6.3 ± 1.8	0.038
HCT, %	39.0 ± 8.6	33.7 ± 8.6	41.6 ± 7.2	40.5 ± 8.3	35.8 ± 8.4	<0.001
LYM, 10^9^/L	1.0 (0.7, 1.4)	0.9 (0.6, 1.2)	1.2 (0.9, 1.7)	1.0 (0.7, 1.4)	0.9 (0.6, 1.3)	<0.001
PLT, × 10^9^/L	213.3 ± 96.8	199.9 ± 113.6	224.1 ± 87.7	215.1 ± 94.3	206.8 ± 99.6	0.001
ALT, U/L	31.0 (16.9, 81.3)	30.9 (15.0, 65.4)	39.0 (20.0, 115.5)	33.0 (17.3, 102.5)	23.0 (14.0, 48.2)	<0.001
AST, U/L	36.0 (23.0, 72.6)	41.6 (25.0, 70.5)	32.1 (21.9, 87.8)	38.3 (23.0, 82.0)	32.0 (21.9, 52.8)	<0.001
TBIL, µmol/L	19.0 (11.8, 33.7)	22.7 (13.0, 50.7)	17.6 (11.3, 27.8)	19.8 (12.4, 35.3)	17.0 (10.6, 28.2)	<0.001
DBIL, µmol/L	6.0 (3.2, 14.0)	9.0 (4.5, 28.4)	4.2 (2.6, 9.0)	6.0 (3.1, 14.3)	6.0 (3.7, 12.4)	<0.001
ALB, g/L	36.1 ± 6.4	28.1 ± 3.3	44.0 ± 4.4	37.5 ± 4.4	30.3 ± 3.1	<0.001
LDH, U/L	376.0 (261.0, 573.0)	441.0 (283.0, 728.0)	274.0 (212.0, 370.0)	376.0 (267.9, 557.2)	455.5 (315.5, 670.0)	<0.001
CK, U/L	74.9 (43.8, 145.0)	82.5 (35.0, 205.1)	75.0 (51.0, 117.8)	74.0 (45.0, 141.0)	70.8 (38.6, 180.0)	0.895
TG, mmol/L	1.9 (1.1, 6.0)	1.8 (1.2, 3.4)	2.3 (1.1, 9.1)	1.8 (1.0, 6.3)	2.1 (1.3, 4.8)	<0.001
TC, mmol/L	4.3 (3.3, 6.1)	3.3 (2.6, 4.4)	5.1 (4.2, 7.3)	4.5 (3.5, 6.4)	3.7 (3.0, 5.1)	<0.001
GLU, mmol/L	9.7 ± 5.4	9.0 ± 4.8	8.8 ± 4.6	10.1 ± 5.7	9.4 ± 5.0	<0.001
LIP, U/L	201.3 (80.0, 537.5)	183.0 (71.9, 439.5)	202.1 (70.8, 486.2)	273.0 (99.0, 673.6)	103.5 (58.5, 247.2)	<0.001
AMY, U/L	238.5 (79.6, 678.2)	161.0 (60.3, 563.2)	203.0 (73.0, 683.6)	310.0 (100.0, 811.8)	128.0 (62.0, 402.0)	<0.001
BUN, mmol/L	5.2 (3.7, 7.4)	6.6 (4.4, 11.4)	4.5 (3.5, 5.9)	5.2 (3.8, 7.1)	5.1 (3.6, 8.0)	<0.001
Cr, µmol/L	67.7 (52.8, 87.5)	76.1 (56.7, 128.3)	64.2 (53.5, 77.6)	68.0 (52.8, 86.8)	66.7 (51.2, 95.2)	<0.001
PaO_2_, mmHg	85.8 ± 27.7	87.1 ± 32.4	88.5 ± 26.0	86.6 ± 26.9	81.6 ± 28.0	<0.001
PaCO_2_, mmHg	33.7 ± 7.3	32.2 ± 8.0	35.9 ± 6.2	33.8 ± 7.1	32.8 ± 7.6	<0.001
Severity scores
SIRS score	1.8 ± 1.1	2.3 ± 1.0	1.3 ± 1.1	1.8 ± 1.1	2.0 ± 1.0	<0.001
APACHE II score	9.8 ± 4.7	12.4 ± 5.1	7.5 ± 3.9	9.6 ± 4.4	10.8 ± 4.7	<0.001
Ranson score	2.0 (1.0, 3.0)	2.0 (1.0, 3.0)	1.0 (0.0, 2.0)	2.0 (1.0, 3.0)	2.0 (1.0, 4.0)	<0.001
BISAP score	1.0 (1.0, 2.0)	2.0 (1.0, 3.0)	1.0 (0.0, 1.0)	1.0 (1.0, 2.0)	2.0 (1.0, 3.0)	<0.001
Specific interventions
ALB infusion within 72 h, *n* (%)	1159 (36.1)	187 (56.2)	67 (13.8)	464 (26.3)	441 (70.2)	<0.001
PCD, *n* (%)	401 (12.5)	51 (15.3)	27 (5.6)	185 (10.5)	138 (22)	<0.001
Mechanical ventilation, *n* (%)	488 (15.2)	100 (30)	23 (4.7)	210 (11.9)	155 (24.7)	<0.001
CRRT, *n* (%)	346 (10.8)	77 (23.1)	17 (3.5)	144 (8.1)	108 (17.2)	<0.001

ALB, albumin; ALT, alanine transaminase; AMY, amylase; AP, acute pancreatitis; APACHE II, acute physiology and chronic health evaluation II; AST, aspartate transaminase; BISAP, bedside index for severity in acute pancreatitis; BMI, body mass index; BUN, blood urea nitrogen; CK, creatine kinase; Cr, creatinine; CRRT, continuous renal replacement therapy; DBIL, direct bilirubin; DBP, diastolic blood pressure; GLU, glucose; Hb, hemoglobin; HbA1c, glycated hemoglobin; HCT, hematocrit; HS-T2, high−stable trajectory2; HTG, Hypertriglyceridemia; LS-T1, low−stable trajectory 1; LI-T4, low−increasing trajectory 4; LDH, lactate dehydrogenase; LIP, lipase; MHF-T3, mid−high−fluctuating trajectory3; NEU, neutrophil; LYM, lymphocyte; PaCO_2_, partial pressure of arterial carbon dioxide; PaO_2_, partial pressure of arterial oxygen; PCD, percutaneous catheter drainage of pancreatic necrosis; PLT, platelet; RANSON, Ranson criteria; SBP, systolic blood pressure; SIRS, systemic inflammatory response syndrome; TBIL, total bilirubin; TC, total cholesterol; TG, triglyceride; WBC, white blood cell.

**Table 2:** Outcomes of the AP patients grouped according to different ALB trajectory

Outcomes	Total (*n* = 3214)	LS-T1 (*n* = 333)	HS-T2(*n* = 486)	MHF-T3 (*n* = 1767)	LI-T4 (*n* = 628)	*P*
OF, *n* (%)	1629 (50.7)	229 (68.8)	120 (24.7)	841 (47.6)	439 (69.9)	<0.001
Respiratory failure, *n* (%	1553 (48.3)	220 (66.1)	117 (24.1)	791 (44.8)	425 (67.7)	<0.001
Renal failure, *n* (%)	455 (14.2)	105 (31.5)	21 (4.3)	202 (11.4)	127 (20.2)	<0.001
Circulatory failure, *n* (%)	255 (7.9)	60 (18)	11 (2.3)	107 (6.1)	77 (12.3)	<0.001
PMOF, *n* (%)	1168 (36.3)	186 (55.9)	69 (14.2)	579 (32.8)	334 (53.2)	<0.001
Pancreatic local complications, *n* (%)	2438 (75.9)	284 (85.3)	242 (49.8)	1363 (77.1)	549 (87.4)	<0.001
APFC, *n* (%)	882 (27.4)	59 (17.7)	117 (24.1)	518 (29.3)	188 (29.9)	<0.001
PPC, *n* (%)	92 (2.9)	16 (4.8)	13 (2.7)	55 (3.1)	8 (1.3)	0.014
WON, *n* (%)	774 (24.1)	130 (39)	54 (11.1)	400 (22.6)	190 (30.3)	<0.001
ANC, *n* (%)	1395 (43.4)	218 (65.5)	100 (20.6)	746 (42.2)	331 (52.7)	<0.001
Severity, *n* (%)	<0.001
MAP, *n* (%)	613 (19.1)	28 (8.4)	215 (44.2)	328 (18.6)	42 (6.7)	–
MSAP, *n* (%)	1433 (44.6)	119 (35.7)	202 (41.6)	860 (48.7)	252 (40.1)	–
SAP, *n* (%)	1168 (36.3)	186 (55.9)	69 (14.2)	579 (32.8)	334 (53.2)	–
IPN, *n* (%)	395 (12.3)	64 (19.2)	24 (4.9)	185 (10.5)	122 (19.4)	<0.001
LOS, days	13.0 (9.0, 21.0)	19.0 (12.0, 32.0)	10.0 (8.0, 13.0)	13.0 (9.0, 20.0)	15.5 (11.0, 26.0)	<0.001
LOS in ICU, days	0.0 (0.0, 7.0)	5.0 (0.0, 16.0)	0.0 (0.0, 0.0)	0.0 (0.0, 5.0)	1.0 (0.0, 10.0)	<0.001
Mortality, *n* (%)	182 (5.7)	59 (17.7)	4 (0.8)	65 (3.7)	54 (8.6)	<0.001

ALB, albumin; ANC, acute necrosis collection; AP, acute pancreatitis; APFC, acute peripancreatic fluid collection; HS-T2, high−stable trajectory2; ICU, intensive care unit; IPN, infected pancreatic necrosis; LI-T4, low−increasing trajectory 4; LOS, length of stay; LS-T1, low−stable trajectory 1; MAP, mild acute pancreatitis; MHF-T3, mid−high−fluctuating trajectory3; OF, organ failure; PMOF, persistent multiple organ failure; PPC, pancreatic pseudocyst; MSAP, moderately severe acute pancreatitis; SAP, severe acute pancreatitis; WON, walled-off necrosis.

### Model development and validation

**Figure 5.**
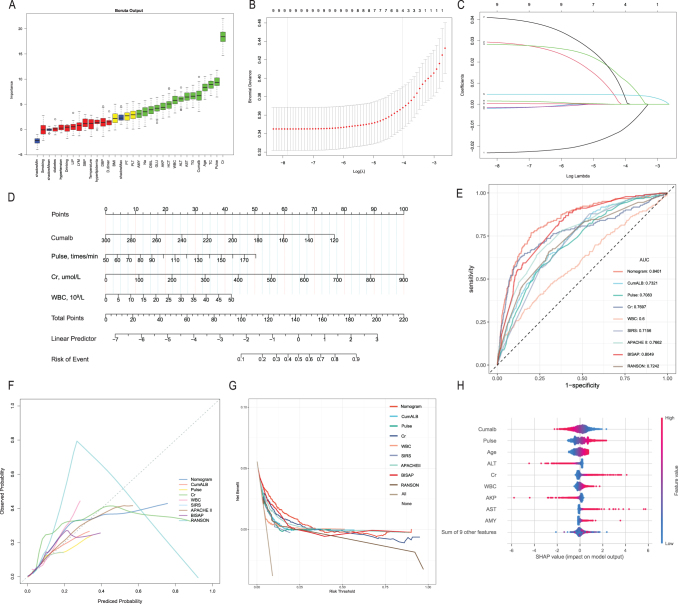
Variable selection, model development, and performance evaluation of the cumalb-based prediction model. **A.** Boruta algorithm output for variable selection, showing importance ranking and classification of variables as confirmed, tentative, or rejected. **B.** SHAP summary plot illustrating the impact and value distribution of each variable on model prediction. **C.** Ten-fold cross-validation for optimal *λ* selection in the LASSO regression model. **D.** Coefficient trajectories of variables under different penalization levels in the LASSO model. **E.** Nomogram incorporating CumALB, pulse, Cr, and WBC for individualized risk estimation. **F.** ROC curves comparing the nomogram, individual predictors, and reference scoring systems. **G.** Calibration plots comparing predicted probabilities and observed outcomes. **H**. Decision curve analysis evaluating the net clinical benefit of each model across varying threshold probabilities. CumALB, cumulative albumin exposure; Cr, serum creatinine; WBC, white blood cell count; SHAP, SHapley Additive exPlanations.

We constructed a machine learning model to predict in-hospital mortality risk in AP patients. Candidate variables were first selected using the Boruta algorithm (Fig. 5A), followed by LASSO regression to optimize model complexity and prevent overfitting by retaining nonzero coefficient variables (Fig. 5B and C, Supplemental Digital Content Table S9, available at, http://links.lww.com/JS9/F9). Four predictors—CumALB, heart rate, Cr, and WBC—were integrated into a logistic regression nomogram (Fig. 5D).

The nomogram achieved an AUC of 0.84, outperforming traditional scoring systems such as APACHE II (AUC = 0.76), SIRS (AUC = 0.72), Ranson (0.72), and BISAP (0.80) (Fig. 5E). Calibration plots and DCA confirmed its reliability and clinical utility, with greater net benefit across risk thresholds, particularly for low-to-moderate-risk patients (Fig. 5F and G). SHAP analysis highlighted CumALB as the most influential predictor (Fig. 5H).

**Figure 6.**
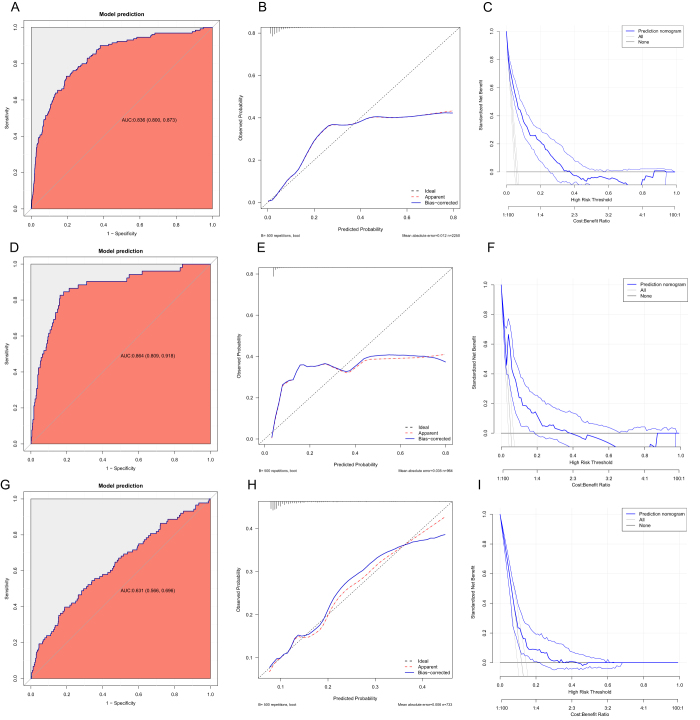
Performance of the CumALB-based prediction model in the training, internal validation, and external validation cohorts. **A**. ROC curve in the training cohort. **B.** Calibration curve in the training cohort. **C.** DCA in the training cohort. **D.** ROC curve in the internal validation cohort. **E.** Calibration curve in the internal validation cohort. **F.** DCA in the internal validation cohort. **G.** ROC curve in the external validation cohort (MIMIC-IV). **H.** Calibration curve in the external validation cohort. **I.** DCA in the external validation cohort. CumALB, cumulative albumin exposure; ROC, receiver operating characteristic; AUC, area under the curve; CI, confidence interval; DCA, decision curve analysis; MIMIC-IV, Medical Information Mart for Intensive Care Iv.

The model performed well during internal and external validation, with satisfactory calibration and clinical usefulness across various thresholds (Fig. 6), with AUCs of 0.836 (95% CI: 0.800–0.873) in the training set (70%) and 0.864 (95% CI: 0.809–0.918) in the test set (30%). External validation in the MIMIC-IV cohort yielded an AUC of 0.631 (95% CI: 0.566–0.696), while further external validation resulted in an AUC of 0.681 (95% CI: 0.532–0.830) in the eICU-CRD cohort and 0.844 (95% CI: 0.789–0.899) in the local cohort from 2024 (Fig. 6, Supplemental Digital Content Fig. S7, available at, http://links.lww.com/JS9/F9). Detailed comparisons of disease severity, demographic characteristics, and treatment strategies across these cohorts are provided in Supplemental Digital Content Table S10, available at, http://links.lww.com/JS9/F9.

### Sensitivity and subgroup analyses

To verify the robustness of the association between CumALB and in-hospital mortality, we performed comprehensive sensitivity analyses addressing potential biases. First, substituting baseline vital signs with cumulative 7-day exposures confirmed consistent associations (adjusted OR = 0.65; 95% CI, 0.49–0.85; Supplemental Digital Content Table S11, available at, http://links.lww.com/JS9/F9). Second, multiple imputation for missing variables showed stable results (adjusted OR = 0.51; 95% CI, 0.42–0.63; Supplemental Digital Content Table S12, available at, http://links.lww.com/JS9/F9). Third, propensity-score matched analysis addressing trajectory-class imbalance reinforced the mortality risk associated with LS-T1 (matched OR = 2.54; 95% CI, 1.56–4.15; Supplemental Digital Content Table S13, available at, http://links.lww.com/JS9/F9, Supplemental Digital Content Fig. S8, available at, http://links.lww.com/JS9/F9). Fourth, analyses based on 72-hour CumALB (adjusted OR = 0.43; 95% CI, 0.35–0.53; Supplemental Digital Content Table S14, available at, http://links.lww.com/JS9/F9) and admission-day albumin (adjusted OR = 0.54; 95% CI, 0.43–0.67; Supplemental Digital Content Table S15, available at, http://links.lww.com/JS9/F9) supported primary findings. Fifth, further adjustment for therapeutic interventions (adjusted OR = 0.63; 95% CI, 0.46–0.85; Supplemental Digital Content Table S16, available at, http://links.lww.com/JS9/F9), temporal analysis excluding early-phase patients (adjusted OR = 0.58; 95% CI, 0.45–0.76; Supplemental Digital Content Table S17, available at, http://links.lww.com/JS9/F9), and exclusion of patients with immunosuppressive diseases or malignancies (adjusted OR = 0.57; 95% CI, 0.44–0.75; Supplemental Digital Content Table S18, available at, http://links.lww.com/JS9/F9) consistently demonstrated robustness. Finally, subgroup analyses confirmed consistent associations across clinical strata without significant interaction (all *P* interaction >0.05; Supplemental Digital Content Fig. S9, available at, http://links.lww.com/JS9/F9).

## Discussion

This study is the first to quantify the risk of in-hospital mortality linked to CumALB over time in AP and to identify four distinct ALB trajectory classes based on dynamic changes during the first 7 days of hospitalization. We demonstrated that CumALB exhibited a linear inverse association with in-hospital mortality and that a predictive model integrating CumALB outperformed traditional scoring systems such as APACHE II and SIRS. Substantial heterogeneity in baseline characteristics and clinical outcomes was observed across ALB trajectory classes. Notably, the LS-T1 group had the highest mortality rate (17.7%), while the LI-T4 group showed significantly lower mortality (8.6%).

By integrating temporal dynamics during the early phase of hospitalization, CumALB addresses limitations of single-time-point measures and provides a more comprehensive quantification of cumulative hypoalbuminemic burden. Multivariable regression analysis showed that for each 1-SD increase in CumALB, the risk of in-hospital mortality decreased by 59% in the unadjusted model and 42% after full adjustment (Model 3). This linear inverse relationship suggests that higher CumALB is associated with lower in-hospital mortality and may serve as a prognostic marker. The findings are consistent with existing literature on time-dependent physiological indices in acute and chronic diseases. For example, Izawa *et al*^[17]^ reported that early cumulative hypotension (mean arterial pressure < 65 mmHg sustained for 3–6 hours) was associated with a 3.73-fold increased risk of progression to severe stages in patients with oliguric acute kidney injury. This underscores the prognostic value of cumulative exposure to physiological derangements.

Similarly, in the cardiovascular field, long-term cumulative blood pressure exposure (e.g., 15-year cumulative systolic/diastolic pressure of 1970.8/1239.9 mmHg · years) was a stronger predictor of disease risk than single-time-point blood pressure measurements, indicating an amplification effect of temporal burden on pathological injury^[19]^. In the pathological context of AP, pancreatic necrosis and enzyme release (e.g., trypsin, elastase) activate systemic inflammation and accelerate ALB depletion^[36]^, a process reflected by CumALB. Of note, the higher proportion of biliary etiology in the low CumALB group suggests that bile reflux may contribute to ALB loss by triggering bile acid–mediated calcium signaling in pancreatic acinar cells, exacerbating both local and systemic inflammation^[37,38]^.

In recent years, numerous studies have confirmed the association between serum ALB levels and outcomes in critically ill patients. However, whether ALB supplementation improves prognosis remains controversial. The latest international guidelines for sepsis management recommend the use of ALB in patients requiring large-volume crystalloid resuscitation^[39]^. A meta-analysis further reported that for every 10 g/L decrease in serum ALB at admission, the risk of mortality increased by 137%, along with significantly prolonged hospital and ICU stays^[40]^. Nevertheless, a randomized controlled trial published in the *New England Journal of Medicine* found no significant difference in 28-day mortality between sepsis patients receiving ALB infusion and those receiving saline. In a subgroup of patients with severe sepsis, ALB treatment showed a trend toward reduced mortality (RR: 0.87, 95% CI: 0.74–1.02), but the difference was not statistically significant^[41]^.

Current fluid resuscitation strategies for AP continue to debate the choice between crystalloids and colloids. While hydroxyethyl starch has been clearly discouraged in existing guidelines^[42,43]^, the role of ALB supplementation remains inadequately addressed^[44]^. A previous small-scale retrospective study from our center suggested that ALB supplementation in patients with baseline hypoalbuminemia could improve prognosis, but dynamic ALB monitoring was not included^[45]^.

Although CumALB offers a means to quantify early ALB burden in AP, it does not fully capture the temporal trajectory of ALB dynamics. To address this, we further utilized LCMM to construct trajectory subtypes, enabling us to evaluate both dynamic patterns and cumulative levels for risk stratification and prognostic assessment. Our findings showed that patients with persistently low ALB trajectories (LS-T1) had the highest mortality rate (17.7%), while those in the low-increasing group (LI-T4) had significantly lower mortality (8.6%), with a 51% reduction in adjusted mortality risk compared to LS-T1. These results align with previous studies in sepsis and ulcerative colitis (UC), where transient hypoalbuminemia followed by early recovery was associated with better clinical outcomes^[13,46]^.In our study, patients with a high-stable trajectory (HS-T2) had markedly better outcomes (mortality 0.8%) than those with moderate-high fluctuating trajectories (MHF-T3; mortality 3.7%). This differs from observations in sepsis, where patients with stable trajectories often had similar outcomes regardless of absolute levels. The discrepancy may stem from the unique pathophysiology of AP, wherein necrotizing inflammation leads to sustained ALB depletion, amplifying the impact of trajectory patterns on outcomes. These findings suggest that dynamic monitoring of both cumulative exposure and ALB trajectories may help resolve current controversies surrounding ALB supplementation in AP—particularly regarding optimal timing and treatment thresholds. Specifically, monitoring dynamic changes in serum albumin within the first week of hospitalization could rapidly inform clinicians about disease severity and may inform risk stratification and monitoring intensity.

Our prediction model, based on the core variable CumALB, was rigorously constructed using both LASSO regression and the Boruta algorithm. Both approaches consistently identified CumALB as a significant predictor, reinforcing the logistic regression results. The model achieved high discrimination in the training cohort (AUC = 0.84), significantly outperforming traditional scoring systems such as APACHE II (AUC = 0.76), SIRS (AUC = 0.72), Ranson (0.72), and BISAP (0.80). In the external MIMIC-IV validation cohort, the model still demonstrated moderate predictive performance (AUC = 0.631). The decrease in external validation performance may be attributed to the inclusion of more severely ill patients from the MIMIC-IV database, and such heterogeneity may have diminished the model’s generalizability. Previous studies have reported higher mortality rates (12.6%–19.3%) in the MIMIC-IV cohort, typically accompanied by more severe organ dysfunction, characterized by lower albumin levels, significantly elevated creatinine levels, and more frequent use of mechanical ventilation and CRRT^[47–49]^. Additionally, regional differences in the etiology of AP^[50,51]^ might further affect disease progression and prognosis, impacting the model’s generalizability. Nonetheless, despite these challenges, our model maintained reasonable robustness and clinical applicability in an external validation cohort characterized by greater disease severity and patient heterogeneity. SHAP value analysis further confirmed the dominant contribution of CumALB to model prediction. Biologically, CumALB has been linked to antioxidant capacity, endothelial stability, and nutritional status^[52,53]^, which may explain its predictive value. The simplified nomogram prediction model constructed in this study, based on dynamic indicators such as CumALB, has the potential to be further developed into automated calculation tools within electronic medical record systems or mobile applications. This would allow clinicians to conveniently calculate and monitor patient mortality risk probabilities, thereby enhancing clinical decision-making efficiency.

This study has several strengths: (1) Compared with previous studies of a similar nature, this study had a larger sample size, providing greater statistical power; (2) To our knowledge, this study is the first to combine CumALB with dynamic ALB trajectory modeling to quantitatively evaluate the associations between time-weighted CumALB burden and dynamic fluctuations of albumin levels with in-hospital mortality in patients with AP; and (3) A simplified logistic regression model based on CumALB, incorporating only four routinely available clinical variables (CumALB, pulse rate, white blood cell count, and serum creatinine) selected via Boruta and LASSO algorithms, provides clinicians with a practical and efficient tool for rapidly and accurately identifying AP patients at risk for mid-to-late in-hospital mortality.

Nonetheless, several limitations should be acknowledged. First, the retrospective cohort spanned approximately 18 years, potentially introducing bias due to evolving clinical practices. However, sensitivity analyses excluding earlier cases demonstrated consistent results, and over 80% of cases were from the recent 9 years. Second, despite validation in three independent cohorts, differences in patient characteristics, disease severity, and management strategies may limit generalizability. Prospective multicenter studies are needed for further validation. Third, stringent inclusion criteria might have introduced selection bias; although we performed extensive sensitivity analyses, residual bias may persist. Finally, given albumin’s long half-life, acute-phase albumin levels may partially reflect premorbid nutritional status. Due to the lack of standardized nutritional assessments, we adjusted for surrogate indicators (e.g., BMI, metabolic history), but residual confounding remains possible.

## Conclusion

This study revealed the prognostic heterogeneity among SAP patients through dynamic monitoring of CumALB and trajectory classification, and developed a machine learning–based predictive tool. Future work should validate this model in prospective multicenter studies and develop targeted intervention strategies to improve clinical outcomes in high-risk populations.

## Data Availability

All data generated or analyzed during this study are included in this published article. Data from public databases can be obtained on the MIMIC - IV website (https://mimic.physionet.org/). Other data in this article can be reasonably applied to the corresponding author.
